# Supplementary material for: Genetic Profiling of Malignant Melanoma Arising from an Ovarian Mature Cystic Teratoma: A Case Report
Source: Int J Mol Sci. 2021 Feb 28;22(5):2436. doi: 10.3390/ijms22052436 (PMC7957566; doi:10.3390/ijms22052436)
Supplement: Supplementary file 1 [file ijms-22-02436-s001.pdf]

TABLE S1. Genes (160) examined in the PleSSision test

ABL1	AKT1	AKT2	ALK	AMER1	APC	AR	ARID1A
ARID2	ASXL1	ATM	ATRX	BAP1	BCL6	BCOR	BRAF
BRCA1	BRCA2	BRIP1	BTK	BUB1B	CARD11	CBL	CBLB
CD79A	CD79B	CDC73	CDH1	CDK12	CDK4	CDKN2A	CHEK2
CIC	CREBBP	CRLF2	CSF1R	CTNNB1	CYLD	DAXX	DDB2
DDR2	DICER1	DNMT3A	ECT2L	EGFR	EP300	EPCAM	ERBB2
ERBB3	ERBB4	ERCC5	ESR1	EZH2	FAM46C	FANCA	FANCD2
FANCE	FAS	FBXO11	FBXW7	FGFR2	FGFR3	FH	FLCN
FLT3	FUBP1	GATA1	GATA2	GATA3	GNA11	GNAQ	GNAS
GPC3	GRIN2A	H3F3A	HIST1H3B	HNF1A	HRAS	HSPH1	IDH1
IDH2	IKZF1	IL6ST	IL7R	JAK1	JAK2	JAK3	KDM6A
KDR	KIT	KLF6	KMT2D	KRAS	MAP2K1	MAP2K2	MAP2K4
MAP3K1	MAP4K3	MDM2	MED12	MEN1	MET	MLH1	MSH2
MSH6	MTOR	MUTYH	MYC	MYD88	NF1	NF2	NFE2L2
NFKBIA	NOTCH1	NOTCH2	NPM1	NRAS	PALB2	PAX5	PBRM1
PDGFRA	PHF6	PIK3CA	PIK3R1	PMS2	PPP2R1A	PRDM1	PRKAR1A
PTCH1	PTEN	PTPN11	RAC1	RB1	RET	ROS1	SDHB
SETD2	SF3B1	SLC7A8	SMAD4	SMARCA4	SMARCB1	SMO	SPOP
SRC	STK11	SUFU	TERT	TNFAIP3	TNFRSF14	TP53	TSC1
TSC2	TSHR	U2AF1	VHL	WT1	XPC	ZNF2	ZRSR2
